# Size of affected vessels in primary angiitis of the CNS: associations with clinical features, medical management and functional outcomes

**DOI:** 10.3389/fneur.2025.1613701

**Published:** 2025-09-17

**Authors:** Franziska Frank, Salomé Jacques, Milani Deb-Chatterji, Alexander Seiler

**Affiliations:** ^1^Department of Neurology, University Hospital Frankfurt, Goethe University, Frankfurt, Germany; ^2^Department of Radiology, Charité - Universitätsmedizin Berlin, Berlin, Germany; ^3^Department of Neurology and Neurovascular Center, University Hospital Schleswig-Holstein, Kiel, Germany

**Keywords:** cerebral vasculitis, primary angiitis of the central nervous system, rare stroke causes, vessel size involvement, immunosuppression

## Abstract

**Background:**

Primary angiitis of the central nervous system (PACNS) presents with heterogeneous clinical manifestations. Since implications of specific patterns of vessel size involvement are not well elucidated, this study aimed to investigate individual disease courses by involved vessel calibers and systematically assess clinical outcomes, relapses, and demographic data.

**Methods:**

This single-center retrospective study (January 2010–April 2022) included 105 cerebral vasculitis patients. PACNS cases (*n* = 49) were stratified into four groups based on radiological vessel involvement: large vessels (Group 1), large and medium-sized vessels (Group 2), small/peripheral vessels (Group 3), and mixed vessel involvement (Group 4). Data on demographics, risk factors, imaging findings, and clinical outcomes were analyzed.

**Results:**

Among 49 PACNS patients (53.1% female, mean age 46.8 years), cardiovascular risk factors, specifically body weight (*p* = 0.021), showed significant differences between groups. Biopsies were positive exclusively in cases with small vessel involvement across all groups (*n* = 9). Cyclophosphamide usage was higher in patients with small vessel involvement (*p* < 0.05). Patients with exclusive small vessel involvement showed greater functional decline (*p* = 0.002 for 2nd relapse), more severe imaging progression (*p* = 0.012 for 3rd relapse) and a trend toward more relapses overall compared to groups without small vessel involvement.

**Conclusion:**

Despite a limited sample size due to the rarity of the disease, our study highlights vessel size as a key factor in PACNS heterogeneity, associating small vessel involvement with worse functional outcomes, greater imaging progression, and distinct treatment patterns. These findings underscore the importance of vessel size in understanding PACNS pathophysiology and guiding management.

## 1 Introduction

Cerebral vasculitis refers to inflammatory processes of the vessel walls in cerebral arteries, capillaries, or venules. This inflammation can lead to complications such as vessel rupture, occlusion, or stenosis, resulting in a range of symptoms from mild to severe. Symptoms include headache, cognitive impairment, seizures, and focal neurological deficits resulting from stroke (1–3). Patients with cerebral vasculitis can be classified into distinct categories based on the underlying etiology: primary, specifically referring to primary angiitis of the central nervous system (PACNS), or secondary, mostly caused by infectious diseases or as cerebral manifestation of systemic vasculitis.

Differentiating between definitive and probable PACNS, as outlined by Birnbaum and Hellmann, is crucial for accurate diagnosis. This differentiation depends on whether the diagnosis is based on biopsy or angiography (4, 5). In the absence of biopsy findings, a diagnosis of probable PACNS may be made based on angiographic evidence of intracranial vessel irregularities typical of vasculitis, in combination with suggestive cerebrospinal fluid (CSF) chemistry, imaging findings, and a compatible clinical syndrome (4). For patients diagnosed via angiography, potential differential diagnoses must be considered. Biopsy remains the gold standard, though reliable biomarkers are lacking (2). It is hypothesized that angiographically diagnosed probable PACNS may have a milder course, though conflicting studies suggest similar or worse outcomes compared to biopsy-confirmed cases (5–8). Notably, there is no clear association between histopathological findings and clinical outcomes, treatment response, or disease progression, underscoring the need for identifying surrogate markers and progression factors.

PACNS has also been divided into two subtypes based on vessel size: large/medium-vessel (LV-PACNS) and small-vessel (SV-PACNS) variants (9). However, definitions of vessel size remain unclear, as medium-vessel cases often also involve small vessels. Nevertheless, the distinction based on vessel size may help with risk stratification and treatment planning. In this retrospective study performed at an academic stroke center, we aimed to characterize individual disease courses in patients with PACNS according to Calabrese and Mallek (8) – including stratification according to the size of vessel involvement. Besides a comprehensive assessment of clinical outcomes and relapses under immunosuppression, we aimed to perform detailed analyses of demographic characteristics and clinical data, available imaging data as well as laboratory findings and vascular risk factors.

## 2 Materials and methods

### 2.1 Study design

**Figure 1:**
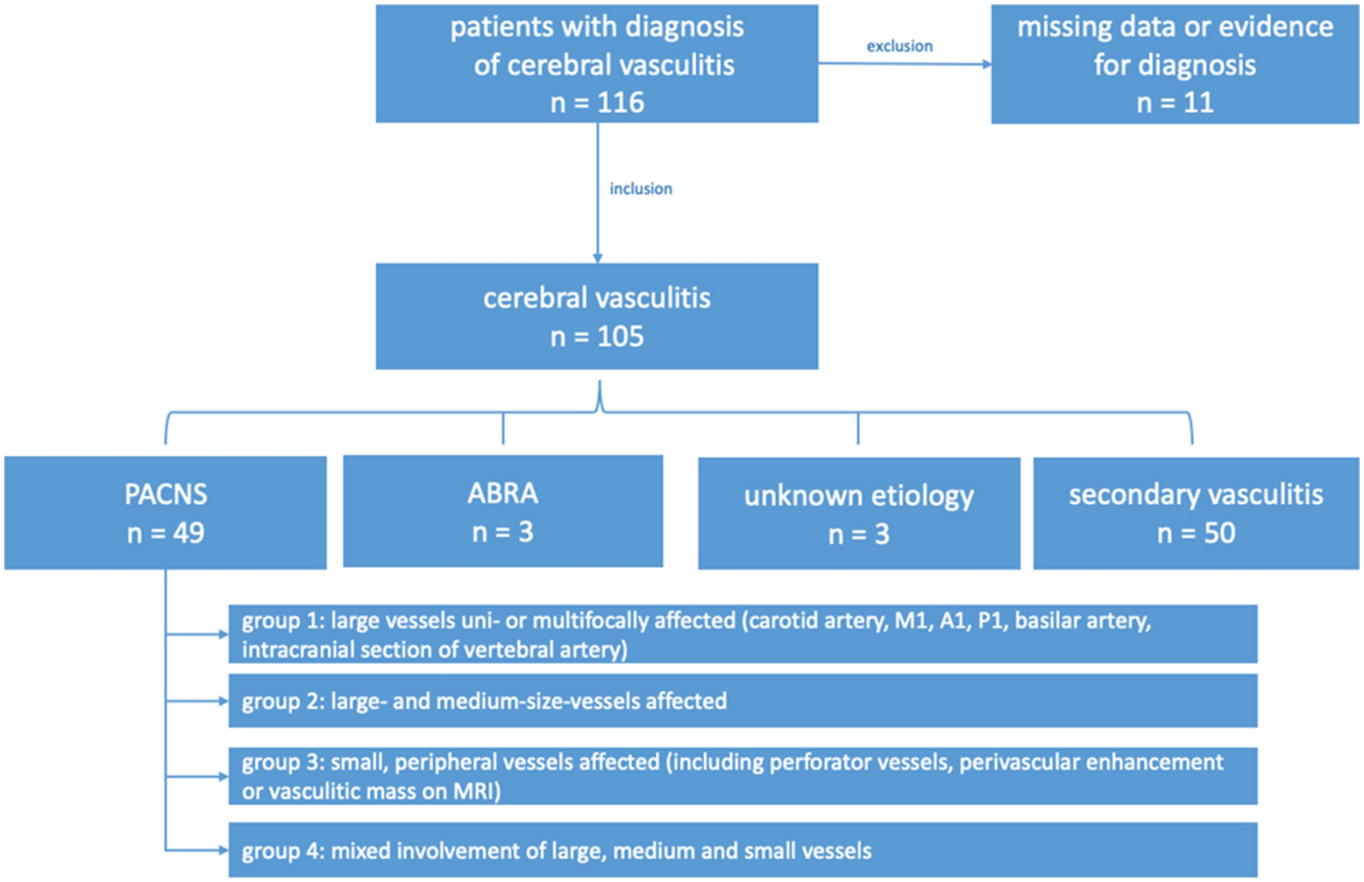
Flow diagram summarizing the inclusion criteria and step-wise inclusion of the study cohort. Out of the initial pool of patients, *n* = 11 were excluded due to insufficient evidence of cerebral vasculitis or missing data. In the middle section, the figure illustrates the classification of patients into four distinct groups based on the etiology of cerebral vasculitis. The group of patients with PACNS was further divided into four subgroups after stratification according to the pattern of vessel involvement. The imaging criteria applied for the subgrouping are outlined in detail in the Materials and Methods section. ABRA, amyloid beta-related angiitis; M1, first segment of the middle cerebral artery; A1, first segment of the anterior cerebral artery; P1, first segment of the posterior cerebral artery; MRI, magnetic resonance imaging.

This retrospective study, conducted at the Department of Neurology, University Hospital Frankfurt, Germany, analyzed demographic, clinical, and radiological data of 105 consecutive patients with a working diagnosis of cerebral vasculitis between January 2010 and April 2022. Case ascertainment used electronic health-record queries of predefined ICD/OPS codes (I63.5, OPS 1–204, I67.7, I67.88, I68.1, I68.2, I77.6) followed by full chart and imaging review; 116 patients were identified and 11 were excluded due to insufficient diagnostic evidence or critical missing data. Patients were subdivided into four groups (Figure 1) by etiology of cerebral vasculitis: primary angiitis of the central nervous system (PACNS), secondary CNS vasculitis, unknown etiology, and amyloid-*β*–related angiitis (ABRA). While the primary focus was PACNS, all cerebral vasculitis patients were initially included for further subdivision. PACNS classification followed Calabrese and Mallek and the Mayo cohort framework: patients were included if biopsy of combined pachy−/leptomeninges with cerebral parenchyma showed histopathology typical of vasculitis, or if angiography and/or vessel-wall imaging demonstrated features consistent with CNS vasculitis (e.g., caliber fluctuations or vessel-wall enhancement). No age cut-off was applied; only incident (first-presentation) cases were included, and patients were drug-naive for vasculitis at index evaluation. In contrast to some prior studies (10), ABRA cases were excluded from PACNS analyses due to presumed distinct pathomechanisms. The study was approved by the Ethics Committee of Goethe University Frankfurt (Faculty of Medicine), which waived the need for informed consent, and all data handling complied with the European General Data Protection Regulation (Art. 30 DSGVO) and the revised Declaration of Helsinki.

### 2.2 Radiological stratification of patients with PACNS

Imaging-based group classification was performed retrospectively. Initial image evaluation was conducted by a neurologist, yielding a preliminary group classification. In a next step, images and group assignments were reviewed by 1–2 neuroradiologists who re-assessed the images independently from the initial rating with consensus adjudication. In cases in which the reviewing neuroradiologists disagreed with the initial assessment, the images were jointly re-evaluated until a consensus was reached. The baseline classification was updated within imaging information of the first year after the initial manifestation if additional vascular territories emerged, which were affected by cerebral vasculitis (Figure 1). These manifestations were assessed using various available imaging techniques such as digital subtraction angiography (DSA), magnetic resonance- or computer tomography angiography (MR-A, CT-A), magnetic resonance imaging (MRI) with contrast agent and black-blood imaging (9, 11), positron emission tomography and computed tomography (PET-CT). Positive biopsy was taken as a proof of small vessel involvement in individual cases.

The distribution of patients within the four groups is as follows:

1. Large vessels, uni- or multifocally affected [internal carotid artery (ICA), M1 segment of the middle cerebral artery (MCA), A1 segment of the anterior cerebral artery (ACA), P1 segment of the posterior cerebral artery (PCA), basilar artery (BA), intracranial section of vertebral artery (VA)], *n* = 9
2. Large and medium-sized vessels (M2-4, A1-4, P1-4 segments) affected, *n* = 13
3. Small or peripheral vessels affected (including perforator vessels, perivascular enhancement or vasculitic tumor-like mass on MRI), *n* = 9
4. Mixed involvement of large, medium and small/peripheral vessels, *n* = 18

### 2.3 Definition framework of key clinical parameters

Age of onset was defined by the first clinical manifestation of cerebral vasculitis. Relapse was identified by the resurgence of symptoms suggestive of vasculitis often supported by imaging findings. Progression in imaging examinations, indicative of a relapses, according to current guidelines, included, e.g., new infarcts, vessel stenosis or vascular caliber fluctuations (9, 12). Functional outcomes were assessed using the modified Rankin Scale (mRS). Cerebrospinal fluid (CSF) analysis was evaluated by two neurologists to identify markers of inflammation. CSF pleocytosis was defined as >4/μl, and protein elevation as >450 mg/L.

### 2.4 Statistical analysis

Statistical analyses were performed using SPSS (IBM, Armonk, NY). For normally distributed continuous variables, t-tests and one-way ANOVA with post-hoc Tukey tests were used. Non-parametric tests (Mann–Whitney U and Kruskal-Wallis) were applied for skewed data, with Dunn’s test for pairwise comparisons. Chi-square tests were used for categorical data. Statistical significance was set at *p* < 0.05. Given the exploratory nature of this study and the rarity of the disease, we did not apply formal correction for multiple testing, as the primary aim was hypothesis generation rather than confirmatory inference. This approach allowed for a more comprehensive evaluation of potential associations in this limited dataset. Heatmaps were created using Inkscape (version 1.2) and Python (version 3.11.5) with the libraries Matplotlib, NumPy, and Pandas.

## 3 Results

### 3.1 Demographic and clinical characteristics of PACNS patients

The cohort of patients with primary angiitis of the central nervous system (PACNS) comprised 46.7% (*n* = 49) of the entire study population (Figure 1). The distribution and frequency, of the various causes of secondary vasculitis as opposed to primary cerebral vasculitis is summarized in the Supplementary Table 1. PACNS patients had a mean age at onset of 46.8 ± 14.2 years, *n* = 26 (53.1%) patients were female. Statistical comparisons of demographic characteristics between the primary and the secondary vasculitis group can be found in Supplementary Table 2. The most frequent symptoms at initial manifestation in PACNS patients were headache (49%), aphasia (38.8%), and motor deficits (36.7%). Detailed clinical manifestations of PACNS at initial presentation are summarized in Supplementary Table 3. The median NIHSS score at initial presentation was 3 (IQR 4), and the median modified Rankin Scale (mRS) score was 2 (IQR 3).

### 3.2 Diagnostic methods and imaging findings for PACNS patients

**Figure 2:**
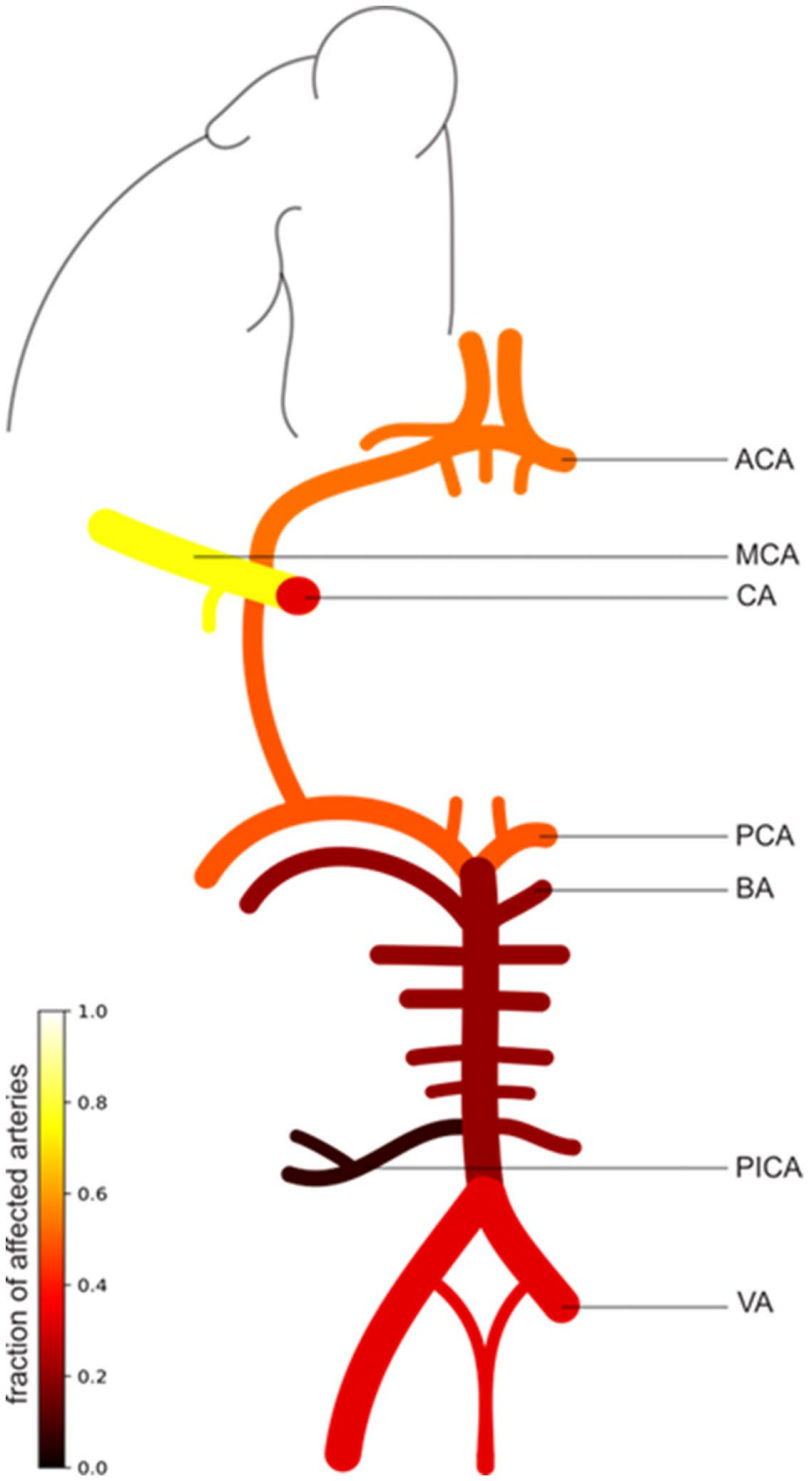
Illustration of the patterns of intracranial vessel involvement in PACNS patients. The scale bar illustrates the fraction of affected arteries (0–1) in all PACNS patients (*n* = 49). A higher fraction and brighter color indicate a greater frequency of involvement. The figure exemplifies only one side, which does not imply that only one side is affected. The MCA was affected in 37 patients (75.5%), the ACA in 26 patients (53.1%), the CA in 16 patients (32.7%), the PCA in 24 patients (49%), the BA in 10 patients (20.4%), the VA in 16 patients (32.7%), and the posterior inferior cerebellar artery (PICA) in 3 patients (6.1%). ACA, anterior cerebral artery; MCA, middle cerebral artery; CA, carotid artery; PCA, posterior cerebral artery; BA, basilar artery; VA, vertebral artery.

The frequencies and results of diagnostic procedures used in PACNS patients in our cohort, with a focus on imaging findings, are presented in Tables 1, 2. Biopsies were performed in 31 out of 49 (63.3%) PACNS cases. Among these biopsies, 9 out of 31 cases (29%) exhibited positive histopathological findings typical of vasculitis and thus were classifiable as definitive PACNS (4). CSF analysis revealed abnormalities (pleocytosis, increased protein levels, oligoclonal bands or cytological results compatible with chronic inflammation) in 32 out of 45 cases analyzed (71.1%). Despite identical proportions for elevated protein levels or leukocyte count, some patients exhibited either elevated protein or CSF pleocytosis. Furthermore, the vast majority of patients diagnosed with PACNS (95.9%, *n* = 47) experienced cerebral infarctions: 89.8% of all PACNS patients experienced ischemic strokes (*n* = 44), and 34.7% intracranial hemorrhage (*n* = 17). Notably, as outlined in Table 1, a significant proportion of all patients suffered from both ischemic and hemorrhagic strokes (28.6%). Assessment of the localization of the cerebral vasculature affected by vasculitis revealed a tendency for greater involvement of the anterior cerebral circulation (87.8% of all cases), as illustrated in Figure 2. The posterior circulation was affected in 71.4% of all cases. Both posterior and anterior cerebral circulation were affected in 65.3% (*n* = 32) of cases, with higher exclusive anterior cerebral circulation involvement (22.4%, *n* = 11) than exclusive posterior cerebral circulation involvement (6.1%, *n* = 3). Details of the diagnostic methods and imaging findings for cerebral vasculitis in the entire cohort are provided in Supplementary Table 4.

**Table 1:** Imaging findings for PACNS patients.

Imaging modalities and findings	PACNS, *n* = 49
Cranial MRI, *n* (%)	49 (100)
Microbleeds	7 (14.3)
All strokes	47 (95.9)
Ischemic stroke	44 (89.8)
Intracranial hemorrhage	17 (34.7)
Ischemic and hemorrhagic stroke	14 (28.6)
Supratentorial stroke	44 (89.8)
Infratentorial stroke	22 (44.9)
Bilateral strokes	29 (59.2)
Contrast-enhanced cranial MRI, *n* (%)	43 (87.8)
Leptomeningeal enhancement	8 (18.6)
Perivascular enhancement	1 (2.3)
Contrast enhancement (vessel wall)	17 (39.5)
Black-blood sequence, *n* (%)	20 (40.8)
MR-angiography, *n* (%)	45 (91.8)
Findings suggestive of vasculitis	34 (75.6)
Digital subtraction angiography, *n* (%)	48 (98)
CT-angiography, *n* (%)	3 (6.1)
PET and CT, *n* (%)	10 (20.4)
Findings suggestive of vasculitis	1 (10)
Caliber fluctuations in any angiography, (*n* %)	41 (83.7)
Doppler- and duplex-ultrasonography, *n* (%)	44 (89.8)
Extracranial atherosclerosis	19 (43.2)
Stenosis	23 (52.3)

PACNS, primary angiitis of the central nervous system; MRI, magnetic resonance imaging; CT, computed tomography; PET, positron emission tomography. In the single case (*n* = 1) with PET/CT, the scan was interpreted as suggestive of vasculitis, showing changes consistent with presumed increased glucose uptake in the walls of both vertebral arteries.

**Table 2:** Radiological stratification based on vessel size involvement in PACNS: demographics and diagnostic findings.

	Patients with PACNS (*n* = 49)	Group 1 (*n* = 9)	Group 2 (*n* = 13)	Group 3 (*n* = 9)	Group 4 (*n* = 18)	*p*-value
Demographics						
Age, mean (SD), years	46.8 (14.2)	37.8 (15.5)	48.9 (10.1)	52.7 (19.5)	46.9 (11.7)	0.139
Women, *n* (%)	26 (53.1)	4 (44.4)	8 (61.5)	3 (33.3)	11 (61.1)	0.472
BMI, mean (SD), kg/m^2^ After exclusion of one outlier	26.3 (5.8) 25.6 (4.0)	26.9 (4.2)	25.5 (3.7)	22.2 (2.7)	29.0 (7.7) 27.3 (3.9)	0.011^*^ 0.018^*^
Weight, mean (SD), kg	78.0 (18.3) 76.1 (13.4)^1^	73.8 (13.4)	75.8 (12.9)	68.0 (14.3)	89.0 (22.7) 83.5 (10.4)^1^	0.021^*^ 0.05^1^
Height, mean (SD), meter	1.72 (0.1)	1.67 (0.1)	1.72 (0.1)	1.74 (0.1)	1.73 (0.1)	0.563
Pack years, mean (SD), years	6.4 (12.4)	3.1 (5.6)	7.9 (15.8)	2.5 (6.1)	9.1 (14.5)	0.803
Current nicotine consumption, *n* (%)	15 (33.3)	5 (55.6)	2 (16.7)	3 (33.3)	5 (33.3)	0.321
Past nicotine consumption, *n* (%)	3 (7.1)	0 (0.0)	1 (9.1)	0 (0.0)	2 (14.3)	0.485
Alcohol abuse, *n* (%)	4 (8.2)	0 (0.0)	2 (15.4)	1 (11.1)	1 (5.6)	0.578
NIHSS at disease onset, median (IQR)	3 (7)	1.5 (8)	4.0 (5)	2.5 (6)	3.0 (12)	0.572
Cardiovascular risk factors and comorbidities						
Hypertension, *n* (%)	24 (49.0)	3 (33.3)	8 (61.5)	4 (44.4)	9 (50.0)	0.618
Diabetes mellitus, *n* (%)	10 (20.4)	2 (22.2)	4 (30.8)	1 (11.1)	3 (16.7)	0.680
Hyperlipidemia, *n* (%)	10 (21.7)	2 (22.2)	2 (15.4)	2 (22.2)	4 (26.7)	0.913
Previous stroke, *n* (%)	6 (12.2)	1 (1.11)	1 (7.7)	3 (33.3)	1 (5.6)	0.192
Diagnostic findings						
CRP, median (IQR), mg/dl	0.33 (0.54)	0.40 (0.47)	0.33 (0.26)	0.67 (0.94)	0.25 (1.06)	0.539
HDL, median (IQR), mg/dl	48.9 (19.0)	49.9 (22.0)	47.7 (26.4)	45.4 (25.8)	52.4 (15.5)	0.717
LDL, median (IQR), mg/dl	102.3 (54.0)	90.7 (40.5)	96.5 (64.0)	103.0 (49.3)	116.0 (79.0)	0.643
triglycerides, median (IQR), mg/dl	117.0 (64.0)	115.0 (68.0)	111.0 (96.0)	109.0 (26.0)	120 (76.0)	0.927
total cholesterol, median (IQR), mg/dl	178.0 (65.0)	156.0 (75.0)	181.0 (45.0)	178.0 (44.0)	205.0 (113)	0.554
HbA1c, median (IQR), %Hb	5.5 (0.7)	5.5 (0.6)	5.7 (2.0)	5.5 (1.0)	5.3 (1.2)	0.414
ESR, median (IQR), mm/h	12.0 (20.0)	7.0 (15.0)	7.0 (26.0)	7.0 (26.0)	16 (23)	0.334
Biopsy, *n* (%)	31 (63.3)	2 (22.2)	9 (69.2)	9 (100.0)	11 (61.1)	0.007^**^
Positive	9 (29.0)	0 (0.0)	0 (0.0)	6 (66.7)	3 (27.3)	0.013^*^
Performed lumbar puncture, *n* (%)	45 (91.8)	8 (88.9)	12 (92.3)	7 (77.8)	18 (100.0)	0.253
Pleocytosis	21 (46.7)	2 (25.0)	5 (41.7)	6 (85.7)	8 (44.4)	0.114
Increased protein levels	21 (46.7)	2 (25.0)	6 (50.0)	4 (57.1)	9 (50.0)	0.583
Cytological results compatible with chronic inflammation	26 (57.8)	4 (50.0)	4 (33.3)	6 (85.7)	12 (66.7)	0.114
Pleocytosis/μl, median (IQR)	26 (62)	13.5 (1)	10 (51)	37.5 (37)	31 (128)	0.246

Variations in the reported numbers and proportions in certain analyses are attributable to missing data. Asterisks denote the level of statistical significance (*p < 0.05). BMI, body-mass-index; mRS, modified Rankin Scale; NIHSS, National Institutes of Health Stroke Scale; CRP, C-reactive protein; HDL, high density; LDL, low density protein; HbA1c, glycated hemoglobin; ESR, erythrocyte sedimentation rate; SD, standard deviation; IQR, interquartile range; kg, kilograms; m, meters; mg, milligrams; dl, deciliters; Hb, hemoglobin; mm, millimeters; h, hours; (1), mean value and standard deviation as well as level of significance after exclusion of one outlier.

### 3.3 Radiological stratification based on vessel size involvement in PACNS

Group comparisons after stratification according to vessel size involvement revealed no significant differences in demographic baseline variables such as age and sex (*p* > 0.05). Upon detailed scrutiny of these four groups, a notable difference in body weight and, consequently, BMI (*p* = 0.011) was observed. Post-hoc tests revealed a significant difference between patients with isolated small vessel involvement and those with mixed vessel involvement, with the latter exhibiting a higher BMI (*p* < 0.001). Patients with affected large vessels showed a higher BMI compared to those exclusively with small vessel involvement (*p* = 0.041), while those with mixed vessel involvement exhibited even higher body weight than those with involvement solely in large vessels (*p* = 0.042). Despite the outlined variations, the demographics, cardiovascular risk factors, and laboratory findings within the four distinct radiological groups did not show any significant differences as demonstrated in Table 2.

In terms of leptomeningeal biopsy, Group 3 exhibited a significantly higher rate of performed biopsies compared to Group 1 (*p* = 0.001) and Group 4 (*p* = 0.03). Conversely, the comparison between Groups 2 and 3 did not reveal any statistically significant differences in the frequency of biopsy performance (*p* = 0.66). Regarding diagnostic outcomes, only Group 3 and 4 - both including cases with small vessel involvement - showed positive biopsy results. Furthermore, Group 1, with exclusive large vessel involvement, demonstrated a significantly lower risk of intracranial hemorrhage compared to Group 2 (*p* = 0.017), Group 3 (*p* = 0.009), and Group 4 (*p* = 0.03). Additionally, Group 1 exhibited less frequent involvement of the vertebrobasilar circulation compared to Group 4, which had mixed vessel involvement (44% vs. 89%, *p* = 0.013). Furthermore, in Group 4 with mixed vessel involvement more findings suggestive of vasculitis were found in MR-angiography than in Group 3 with only small vessel involvement (*p* = 0.020). More specifically, patients in Group 4 showed a higher frequency of caliber fluctuations on cerebral angiograms compared not only to Group 3, which included small vessel involvement (*p* = 0.009), but also to Group 1, characterized by exclusive large vessel involvement (*p* = 0.009). Significant differences were observed in the prevalence of vascular stenoses between Group 1 (88.9%), characterized by large-vessel involvement, and both Groups 2 (36.4%) and Group 3 (12.5%) as well as Group 4 (62.5%) compared to Group 3 (Group 1 vs. Group 2: *p* = 0.017; Group 1 vs. Group 3: *p* = 0.002; Group 4 vs. Group 3: *p* = 0.02).

Regarding functional outcomes, there were no significant differences between the four groups in terms of the number of relapses or the absolute mRS scores before or after relapses, as demonstrated in Table 3. Nevertheless, the difference between absolute mRS scores before and after relapse was calculated for all relapses and groups: For the second relapse, Group 3, consisting of patients with exclusive small vessel involvement, experienced a significantly higher increase of the mRS score and thus more pronounced worsening of functional status, when compared to Groups 1 (*p* = 0.046), 2 (*p* = 0.014), and 4 (*p* = 0.003). Group 3 was also the only group for which an mRS difference before and after the second relapse was noticed at all. Furthermore, upon detailed examination of the third relapse, Group 2 exhibited a lower frequency of progression in imaging markers compared to both Group 3 (*p* = 0.046) and Group 4 (*p* = 0.014), each characterized by the involvement of small vessels.

**Table 3:** Radiological stratification based on vessel size involvement in PACNS: outcome and follow-up.

	Patients with PACNS (*n* = 49)	Group 1 (*n* = 9)	Group 2 (*n* = 13)	Group 3 (*n* = 9)	Group 4 (*n* = 18)	*p*-value
mRS before disease onset, median (IQR)	0 (1)	0 (1)	0 (2)	0 (1)	0 (1)	0.470
mRS after initial manifestation of disease, median (IQR)	2 (3)	1 (2)	3 (2)	2 (3)	2 (4)	0.146
Follow-up, median (IQR), years	2.0 (4.9)	1.5 (7.0)	2 (1.4)	1.0 (5.6)	2.2 (4.7)	0.980
Relapses, *n* (%)	33 (67.3)	5 (55.6)	8 (61.5)	8 (88.9)	12 (66.7)	0.779
number of relapses, median (IQR)	1 (2.0)	1 (2)	1 (2)	1 (3)	1 (3)	0.765
Maintenance therapy with cyclophosphamide, *n* (%)	31 (63.3)	3 (33.3)	6 (46.2)	7 (77.8)	15 (83.3)	0.029^*^
mRS before relapse 1, median (IQR)	2 (2)	0.5 (3)	2 (3)	3 (2)	1.5 (2)	0.497
mRS after relapse 1, median (IQR)	3 (4)	3 (5)	3 (4)	4 (3)	3 (4)	0.873
Relapse 1 under maintenance therapy, *n* (%)	9 (27.3)	1 (20.0)	2 (25.0)	3 (37.5)	3 (25.0)	0.798
Relapse 1 under cortisone therapy, *n* (%)	9 (27.3)	0 (0.0)	2 (25.0)	2 (25)	5 (41.7)	0.379
mRS before relapse 2, median (IQR)	1.5 (2)	2.5 (5.0)	1.5 (3)	2 (3.0)	1 (2)	0.993
mRS after relapse 2, median (IQR)	1.5 (2.75)	2.5 (5.0)	1.5 (3)	3 (3.0)	1 (2.0)	0.934
Relapse 2 under maintenance therapy, *n* (%)	6 (37.5)	0 (0.0)	3 (75.0)	1 (33.3)	2 (28.6)	0.277
Relapse 2 under cortisone therapy, *n* (%)	5 (31.3)	0 (0.0)	2 (50.0)	1 (33.3)	2 (28.6)	0.661
mRS before relapse 3, median (IQR)	2 (2.0)	2 (4.0)	1	3 (3)	2 (2)	0.721
mRS after relapse 3, median (IQR)	3 (3.0)	2 (4.0)	1	4 (0)	3 (2)	0.220
Relapse 3 under maintenance therapy, *n* (%)	8 (72.7)	1 (50.0)	0 (0.0)	3 (100.0)	4 (80.0)	0.217
Relapse 3 under cortisone therapy, *n* (%)	7 (63.6)	1 (50.0)	0 (0.0)	3 (100.0)	3 (60.0)	0.301

Asterisks denote the level of statistical significance (*p < 0.05). mRS, modified Rankin Scale; IQR, interquartile range.

### 3.4 Management and therapy

Alongside glucocorticoids, cyclophosphamide (CYC) was most frequently administered as immunosuppressant in PACNS patients, with 63.2% of cases after initial manifestation of the disease. At our center, cyclophosphamide usually follows the nadir-guided Austin protocol (15–20 mg/kg i.v. every 4 weeks, adjusted to body surface area and leukocyte nadir) and glucocorticoid induction consists of 3–5 days i.v. methylprednisolone (up to 1 g/day) followed by a body-weight–adjusted oral taper (13). Other immunosuppressive medications employed in our cohort of PACNS patients included Rituximab (RTX), Azathioprine (AZA), and Mycophenolate Mofetil (MMF) (see Supplementary Table 5). Therapeutic regimens and dosing were individualized according to clinical context; consequently, not all patients received the same treatment. CYC was significantly more frequently administered in patients with small vessel involvement (Groups 3 and 4). Specifically, Group 4 (mixed vessel involvement) exhibited a significantly higher rate of usage compared to Group 1 (*p* = 0.009) and Group 2 (*p* = 0.029), alongside a trend toward greater usage in Group 3 relative to Group 1 (*p* = 0.058). Notably, AZA was only applied in Group 1, while Group 1 was the only group in which no patient received RTX as a treatment.

Regarding relapses, there was no significant difference in frequency when comparing the four groups (*p* = 0.078), but a tendency toward higher percentages of relapses in the groups with small vessel involvement (see also Supplementary Table 6). In terms of glucocorticoid treatment, Group 1, with large vessel involvement only, was less frequently administered corticosteroids at the time of the second relapse compared to Group 2 (*p* = 0.014) and Group 3 (*p* = 0.025). Additionally, when examining the timepoint of the third relapse, Group 2 with large and medium vessel involvement exhibited a longer therapy-free interval compared with Group 4 (*p* = 0.025). Furthermore, Group 2 was less frequently under immunosuppressive therapy compared to Group 4 for the fourth relapse (*p* = 0.025).

## 4 Discussion

In this retrospective study conducted at an academic stroke center, we aimed to characterize the disease courses of PACNS patients, stratified by vessel size involvement, through comprehensive analyses of clinical outcomes, relapses, demographic characteristics, imaging, laboratory findings, and vascular risk factors.

In the PACNS cohort, 53.1% of patients were female, suggesting a nearly even sex distribution across affected patients, which is supported by recent meta-analyses (14). The average age at onset was 46.8 years, in line with previous reports from the literature (2, 15, 16). Among the 49 PACNS patients assessed, 49% had hypertension and 20% had diabetes. Considering the mean age of the cohort, these percentages are noteworthy and exceed typical prevalence rates for this age group. For example, the prevalence of hypertension in 45–64 year olds ranges from 31.6% in women to 38.3% in men (17). Prevalence of diabetes mellitus generally does not exceed 10% (18, 19). These findings indicate a significantly higher percentage of cardiovascular risk factors in PACNS patients compared to the normal population of the same age group, suggesting that the pathophysiology of PACNS is more complex and may require targeting risk factors alongside immunosuppressive strategies. Notably, 71.1% of patients showed inflammatory CSF results (median cell count of CSF pleocytosis: 26/μl, IQR: 62), which supports the PACNS diagnosis and argues against other important differential diagnoses of cerebral vasculitis, such as intracranial atherosclerosis. Referring to Ramirez-Lassepas et al., acute stroke patients less frequently exhibit pleocytosis (12.7%), with the highest cell counts reaching only 15/μl (20). Abnormal CSF findings (71.1%) in our study are comparable to findings of previous research, which reported inflammatory findings in CSF (pleocytosis or elevated protein concentration) in 65–90% of PACNS cases (10, 16).

Most prevalent clinical symptoms in our cohort included motor deficits (36.7%), aphasia (38.8%), and headache (49%). Compared to the Mayo cohort (hemiparesis: 40.5%; aphasia: 24.5%; headache: 59.5%), our study showed similar rates for motor deficits but a 10% lower incidence of headaches. However, aphasia was more pronounced in our cohort, potentially due to a higher incidence of stroke (10). A significant proportion of PACNS patients in our study experienced cerebral infarctions (95.9%), which is substantially higher compared to 54% found in the Mayo Clinic Cohort (10). In their patient collective there was a higher proportion of cerebral infarcts in angiography-confirmed patients compared to biopsy-confirmed cases, which may explain the disparity with our study (21), where 18.4% were biopsy-confirmed (29% of all cases where biopsy was conducted) versus 35.6% in the Mayo Cohort (10). Although the percentage of bilateral infarcts in our study is similar to angiographically confirmed cases from the Mayo cohort, our cohort had a higher prevalence of intracranial hemorrhages (34.7% vs. 10.8%). Notably, 28.6% of patients suffered both ischemic and hemorrhagic infarctions, highlighting the importance of recognizing the co-occurrence of these conditions as strongly indicative of PACNS.

Our study design, which stratifies PACNS patients into four radiologically defined groups, may provide a more precise approach compared to the traditional, most likely oversimplified distinction between SV-PACNS and LV-PACNS (9). Although the overall age distribution did not show significant differences, a trend suggested younger ages among individuals with large vessel involvement, contrasting with de Boysson et al., who found a younger age profile among patients with small vessel involvement (16). Regarding cardiovascular risk factors, patients with large or mixed vessel involvement tended to have a higher BMI compared to those with small vessel involvement. Those with mixed vessel involvement exhibited even higher weight than those with solely large vessel involvement. A significantly higher percentage of patients with small vessel involvement underwent biopsy compared to those with large or mixed vessel involvement. This may be due to the lower diagnostic yield expectations in cases with large or mixed vessel involvement (9). Notably, biopsies in all four groups were positive only when small vessels were involved. This supports the current clinical practice of performing biopsies primarily in SV-PACNS patients, and if mixed vessels are involved, biopsy should be considered. Diagnostic results showed more stenoses in groups with large vessel involvement, but significantly fewer intracranial hemorrhages compared to the other groups. Study results focusing on the influence of vessel size and the differential impact on anterior versus posterior circulation are currently lacking. Our study demonstrates that, compared to large vessel involvement, the posterior circulation is more frequently affected in patients with mixed vessel involvement. The reasons for this finding remain currently unknown. However, it could hold significant prognostic value and influence clinical decision-making with regard to which patients should be selected for biopsy.

Concerning functional outcomes according to affected vessel sizes in PACNS, our results are in line with findings from previous studies indicating more unfavorable long-term outcomes in patients with small vessel involvement (22) compared to patients with large- or medium-sized vessels affected (Group 1 and 2). This hypothesis is supported by a higher imaging progression rate at the third relapse (Groups 3 and 4), more unfavorable outcomes at the second relapse point for Group 3, as well as longer therapy-free intervals of Group 2 compared to Group 4. Furthermore, our study suggests that patients in our cohort with small vessel involvement are treated more aggressively, reflected by the higher frequency of CYC use in Groups 3 and 4 compared to Groups 1 and 2. AZA, less potent in immunosuppression than RTX or CYC, was only given in Group 1, while Group 1 was the only group in which RTX was not used. Additionally, Group 1 and 2 patients received immunosuppressive therapy, including glucocorticoids, less frequently compared to the other groups at different relapse points. The higher rate of glucocorticoid usage in groups with small vessel involvement contrasts with the treatment recommendations in the Mayo cohort (10).

### 4.1 Limitations

This study has several strengths, including comprehensive data analysis in patients with a rare condition and a large dataset from a single center with a broad catchment area, representing a diverse patient population. However, it also has certain limitations that need to be acknowledged. The small sample size, inherent to the rarity of the disease, limits the generalizability of our findings – especially with regard to a comparison of relapses in subgroup analyses. Furthermore, only 18.4% of the study population had biopsy-proven diagnoses (excluding ABRA cases), potentially affecting the accuracy of our conclusions. Additionally, the retrospective design of this study carries the possibility of unknown sources of bias. Because follow-up imaging was symptom-triggered and not always pre-planned or obtained at fixed intervals, variable timing may have biased detection of radiographic progression, involvement of vascular territories beyond the initial pattern of affected vessel calibers later in the course of the disease and the dating of relapses. These points reflect inherent limitations of the retrospective design of our study. As treatment choice, dosing, and duration were individualized in this retrospective cohort without a uniform protocol, heterogeneity in immunosuppressive regimens and undocumented therapy changes during follow-up may limit comparability of treatment effects between patients and subgroups stratified according to the size of affected vessels. It is important to note that prospective data is scarce in the field of PACNS, limiting opportunities for comparison. These factors should be considered when interpreting the results and their applicability to wider clinical practice.

## 5 Conclusion

Despite the limited sample size, the results of our study suggest distinct differences in cardiovascular risk factors across the four groups defined by the size of affected vessels in PACNS. Small vessel involvement was linked to more severe functional decline, greater progression of imaging markers and a higher rate of CYC usage. Biopsies were exclusively positive in cases in which small vessels are affected, underscoring the importance of prioritizing biopsies in SV-PACNS patients as well as in cases with mixed vessel involvement. Furthermore, patterns of macrovascular and medium-vessel involvement on angiography might provide some indication concerning the expected diagnostic yield of biopsy in individual cases. Taken together, our results suggest that, besides differentiating biopsy-proven and angiographically confirmed PACNS cases, further subgrouping based on vessel size involvement might become increasingly important for accurate risk stratification and the development of specific therapeutic strategies.

## Data Availability

The original contributions presented in the study are included in the article/Supplementary material, further inquiries can be directed to the corresponding author.
