# Supplementary material for: Size of affected vessels in primary angiitis of the CNS: associations with clinical features, medical management and functional outcomes
Source: Front Neurol. 2025 Sep 17;16:1613701. doi: 10.3389/fneur.2025.1613701 (PMC12483884; doi:10.3389/fneur.2025.1613701)
Supplement: Supplementary file 1 [file Table_1.docx]

Supplementary Material

# Supplementary Tables

**Supplemental Table 1.** Distribution of identified causes for secondary vasculitis in the entire patient cohort.

| Secondary vasculitis (n=50) | | |
| --- | --- | --- |
| **Infectious Vasculitis** | **n = 28 (56%)** | |
|  | HIV | 8 (16%) |
|  | Streptococcus pneumoniae | 6 (12%) |
|  | Tuberculosis | 3 (6%) |
|  | Syphilis | 3 (6%) |
|  | VZV | 3 (6%) |
|  | Nocardia | 1 (2%) |
|  | Hepatitis C | 1 (2%) |
|  | Borreliosis | 1 (2%) |
|  | Fusobacterium | 1 (2%) |
|  | Unknown | 1 (2%) |
| **Systemic Vasculitis** | **n = 12 (24%)** | |
|  | Giant cell arteriitis | 5 (10%) |
|  | Granulomatosis with polyangiitis (GPA) | 2 (4%) |
|  | Behçet Disease | 2 (4%) |
|  | Eosinophilic granulomatosis with polyangiitis (EGPA) | 1 (2%) |
|  | Diagnosis cannot be specified | 2 (4%) |
| **Vasculitis in connection with other autoimmune disease** | **n = 8 (16%)** | |
|  | Sarcoidosis | 3 (6%) |
|  | Sharp syndrome | 2 (4%) |
|  | Crohn’s disease | 1 (2%) |
|  | Familial mediterranean fever | 1 (2%) |
|  | Unknown autoimmune disease | 1 (2%) |
| **Drug -induced Vasculitis** | **n = 2 (4%)** | |
|  | Nivolumab | 1 (2%) |
|  | Etanercept | 1 (2%) |

The majority of cases (56%) were attributed to infectious vasculitis, followed by 24% related to systemic vasculitis. Additionally, 16% of cases were associated with other autoimmune diseases, while 4% were drug-induced

**Supplemental Table 2.** Demographic and clinical baseline characteristics along with laboratory findings for all patients with primary and secondary cerebral vasculitis.

|  | Secondary cerebral vasculitis (n=50) | PACNS (n=49) | | p-value |
| --- | --- | --- | --- | --- |
| Demographics |  |  | |  |
| age, mean (SD), years | 51.7 (14.5) | 46.8 (14.2) | | 0.848 |
| women, n (%) | 18 (36) | 26 (53.1) | | 0.088 |
| weight, mean (SD), kg | 74.1 (20.6) | 78(18.3) | | 0.314 |
| height, mean (SD), m | 1.7 (0.1) | 1.7 (0.1) | | 0.757 |
| BMI, mean (SD), kg/m2 | 24.6 (6) | 26.3 (5.8) | | 0.069 |
| mRS after disease onset, median (IQR) | 2.0 (3.0) | 2.0 (3.0) | | 0.418 |
| NIHSS after disease onset, median (IQR) | 3.0 (4.0) | 3.0 (7.0) | | 0.646 |
| pack years, mean (SD), years | 13.7 (17.8) | 6.4 (12.4) | | 0.117 |
| current nicotine consumption, n (%) | 12 (33.3) | 15 (33.3) | | 1.000 |
| past nicotine consumption, n (%) | 8 (22.9) | 3 (7.1) | | 0.050 |
| follow-up, median (IQR), years | 0.5 (5.6) | 2.0 (4.9) | | 0.062 |
| relapses, n (%) | 12 (24) | 32 (65.3) | | <0.001^***^ |
|  |  |  | |  |
| Cardiovascular comorbidities, n (%) | |  |  |  |
| hypertension | 22 (44) | 24 (49) | | 0.619 |
| diabetes mellitus | 12 (24) | 10 (20.4) | | 0.667 |
| coronary artery disease | 1 (2) | 2 (4.1) | | 0.558 |
| hyperlipidemia | 12 (27.9) | 10 (21.7) | | 0.500 |
| previous stroke | 4 (8) | 6 (12.2) | | 0.483 |
| hypertensive heart disease | 1 (2) | 1 (2) | | 0.988 |
| atrial fibrillation | 4 (8) | 2 (4.1) | | 0.414 |
| estrogen-containing drugs | 1 (2) | 2 (4.1) | | 0.546 |
| sleep apnea | 2 (4) | 1 (2) | | 0.570 |
| kidney disease | 4 (8) | 1 (2) | | 0.176 |
| Diagnostic findings |  |  | |  |
| CRP, mean (SD), mg/dl [< 0.5] | 1.4 (7.7) | 0.33 (0.54) | | 0.006^**^ |
| HDL, median (IQR), mg/dl [> 40] | 39.9 (22.1) | 48.9 (19.0) | | 0.227 |
| LDL, median (IQR), mg/dl [< 115] | 82.5 (39.8) | 102.3 (54.0) | | 0.242 |
| triglycerides, mean (SD), mg/dl [< 325] | 137.0 (88.0) | 117.0 (64.0) | | 0.277 |
| total cholesterol, mean (SD), mg/dl [< 190] | 161.0 (70.0) | 178.0 (65.0) | | 0.250 |
| HbA1c, mean (SD), %Hb [4.8-5.9] | 5.9 (1.2) | 5.5 (0.7) | | 0.146 |
| ESR, mean (SD), mm/h [< 20] | 32.0 (38.0) | 12.0 (20.0) | | 0.009^**^ |
| biopsy, n (%) | 13 (26) | 31 (63.3) | | <0.001^***^ |
| - positive | 10 (76.9) | 9 (29) | | 0.003^**^ |
| performed lumbar puncture, n (%) | 48 (96) | 45 (91.8) | | 0.385 |
| - pleocytosis | 33 (68.8) | 21 (46.7) | | 0.031^*^ |
| - increased protein levels | 32 (66.7) | 21 (46.7) | | 0.052 |
| - cytological results compatible with chronic inflammation | 22 (45.8) | 26 (57.8) | | 0.249 |
| - pleocytosis/µl, median (IQR) | 40 (153) | 26 (62) | | 0.291 |

Variations in the reported numbers and proportions in certain analyses are due to missing data. The criteria for evaluating lumbar puncture results are detailed in the Materials and Methods section. BMI: body-mass-index; mRS: modified ranking scale; NIHSS: National Institutes of Health Stroke Scale; CRP: C-reactive protein; HDL: high density; LDL: low density protein; HbA1c: glycated hemoglobin; ESR: erythrocyte sedimentation rate; SD: standard deviation; kg: kilograms; m: meters; mg: milligrams; dl: deciliters; Hb: hemoglobin; mm: millimeters; h: hour; µl: microliters; IQR: interquartile range.

**Supplemental Table 3.** Distribution of symptoms observed at initial diagnosis of cerebral vasculitis for the PACNS group.

|  |  | PACNS (n=49) |
| --- | --- | --- |
| Cognition | Cognitive decline | 1 (2.0%) |
|  | Attention deficit | 3 (6.1%) |
|  | Memory disorder | 4 (8.2%) |
| Psychiatric symptoms | Personality change | 4 (8.2%) |
|  | Confusion | 1 (2.0%) |
|  | Depressive symptoms | 1 (2.0%) |
|  | Psychomotor impairment | 1 (2.0%) |
| Impairments in  vigilance | Impaired vigilance | 2 (4.1%) |
|  | Coma | 1 (2.0%) |
|  | Status epilepticus | 2 (4.1%) |
| Neurological deficits | Visual impairment | 2 (4.1%) |
|  | Scotoma | 1 (2.0%) |
|  | Oculomotor disorder | 3 (6.1%) |
|  | Ptosis (Horner’s syndrome) | 1 (2.0%) |
|  | **Motor deficits** | **18 (36.7%)** |
|  | Fine motor skills disorders | 3 (6.1%) |
|  | Sensory disturbances | 9 (18.4%) |
|  | Coordination disorders | 1 (2.0%) |
|  | **Aphasia** | **19 (38.8%)** |
|  | Dysarthria | 3 (6.1%) |
|  | Dizziness | 4 (8.2%) |
|  | Gait unsteadiness/ Gait disorders | 3 (6.1%) |
| General non-specific symptoms | **Headaches** | **24 (49.0%)** |
|  | Fatigue, worsening of general condition | 2 (4.1%) |
|  | Fever | 1 (2.0%) |
|  | Nausea/Vomiting | 3 (6.1%) |

Symptoms are categorized into cognition, psychiatric symptoms, impairments in vigilance, neurological deficits, general non-specific symptoms and indications of past infection. The table highlights that headache is the most common symptom overall, followed by motor deficits and aphasia.

| **Supplemental Table 4.** Diagnostic methods and imaging findings for cerebral vasculitis in the entire cohort.   \| **Diagnostic method**   - imaging findings \| Cerebral vasculitis  n = 105 \| \| --- \| --- \| \| **Cranial MRI, (n %)** \| 103 (98.1) \| \| - microbleeds \| 18 (17.5) \| \| - ischemic stroke \| 85 (81) \| \| - intracranial hemorrhage \| 24 (22.9) \| \| **Contrast-enhanced cranial MRI, n (%)** \| 95 (90.5) \| \| - leptomeningeal enhancement \| 21 (22.1) \| \| - perivascular enhancement \| 8 (8.4) \| \| - contrast enhancement (vessel wall) \| 28 (29.5) \| \| **Black-blood sequence, n (%)** \| 29 (27.6) \| \| **MR-angiography, n (%)** \| 91 (86.7) \| \| - findings suggestive of vasculitis \| 66 (72.5) \| \| **Digital subtraction angiography, (n %)** \| 80 (76.2) \| \| **CT- angiography, (n %)** \| 7 (6.7) \| \| **PET and CT, n (%)** \| 18 (17.1) \| \| - findings suggestive of vasculitis \| 2 (11.1) \| \| **Caliber fluctuations in any angiography, (n %)** \| 74 (72.5) \| \| **Doppler- and Duplex-ultrasonography, n (%)** \| 93 (88.6) \| \| - extracranial atherosclerosis \| 32 (34.4) \| \| - stenosis \| 48 (51.6) \| \| **Biopsy, n (%)** \| 47 (44.8) \| \| - vasculitis typical findings \| 22 (46.8) \| |
| --- | --- | --- | --- | --- | --- | --- | --- | --- | --- | --- | --- | --- | --- | --- | --- | --- | --- | --- | --- | --- | --- | --- | --- | --- | --- | --- | --- | --- | --- | --- | --- | --- | --- | --- | --- | --- | --- | --- | --- | --- | --- | --- | --- | --- |

MRI: Magnetic Resonance Imaging; CT: Computed Tomography; PET: positron emission tomography.

**Supplemental Table 5.** Immunosuppressive therapy after the initial manifestation in the PACNS group.

|  | PACNS (n=49) |
| --- | --- |
| Cyclophosphamide | 31 (63,2%) |
| Mycophenolate Mofetil | 12 (24,5%) |
| Rituximab | 5 (10,2%) |
| Azathioprine | 2 (4,1%) |

The table highlights induction immunosuppressive therapies, with cyclophosphamide being the most common therapy after initial manifestation. Dosing at our center follows standard protocols: cyclophosphamide per the Austin regimen (15–20 mg/kg i.v. every 4 weeks, BSA- and leukocyte-nadir–adjusted) and mycophenolate mofetil 2 g/day; azathioprine and rituximab were used in selected cases per standard clinical practice. Owing to the retrospective, non-protocolized design, dose adjustments cannot be excluded. Glucocorticoids are excluded from this table and are instead detailed in Table 3 of the main manuscript.

**Supplemental Table 6.** Mean time intervals (standard deviation) in months until the first, second and third relapse in the different PACNS subgroups stratified according to vessel size involvement .

| number of relapses for different time points^1-3^ | PACNS total  n^1^ = 33  n^2^ = 16  n^3^ = 11 | Group 1  n^1^ = 5  n^2^ = 2  n^3^ = 2 | Group 2  n^1^ = 8  n^2^ = 4  n^3^ = 1 | Group 3  n^1^ = 8  n^2^ = 3  n^3^ = 3 | Group 4  n^1^ = 12  n^2^ = 7  n^3^ = 5 |
| --- | --- | --- | --- | --- | --- |
| average time until first relapse in months, mean (SD) | 7 (15.5) | 6.6 (9.9) | 4.5 (8) | 13.6 (2.6)  3.5 (28.6)^4^ | 4.5 (7.5) |
| average time until second relapse  in months, mean (SD) | 8.7 (17.5) | 6 (2.8) | 21.6 (33.8) | 3.9 (3.8) | 4.1 (6.2) |
| average time until third relapse  in months, mean (SD) | 15.5 (27.2) | 9 (0) | 96 | 6.2 (3.8) | 7.5 (7.2) |

Note 1-3 indicate different n-numbers depending on the relapse 1-3, note 4 indicates the mean time interval obtained after exclusion of an outlier.
